# Supplementary material for: Mutual Associations of Healthy Behaviours and Socioeconomic Status with Respiratory Diseases Mortality: A Large Prospective Cohort Study
Source: Nutrients. 2023 Apr 13;15(8):1872. doi: 10.3390/nu15081872 (PMC10142455; doi:10.3390/nu15081872)
Supplement: Supplementary file 1 [file nutrients-15-01872-s001.zip › nutrients-2241101-supplementary.pdf]

Supplementary appendix

This appendix formed part of the original submission.

Appendix method. Assessment of socioeconomic status using latent class analysis 2

Table S1. The numbers (percentages) of participants with missing covariates (N=542,414) 5

Table S2. Scoring system for healthy behaviour indices. 6

Table S3. Sensitivity analyses of associations between socioeconomic status with respiratory disease mortality. 7

Table S4. Associations between socioeconomic status with mortality from influenza and pneumonia and chronic lower respiratory diseases. 8

Figure S1. Associations of healthy behaviors or socioeconomic status with respiratory diseases mortality. 9

Table S5. Associations of socioeconomic status with mortality from influenza and pneumonia and chronic lower respiratory diseases by healthy behaviors. 10

Table S6. Associations of healthy behaviours with respiratory disease mortality. 11

Table S7. Associations of healthy behaviors with mortality from influenza and pneumonia and chronic lower respiratory diseases by socioeconomic status. 12

Table S8. Joint associations of healthy behaviors and socioeconomic status with respiratory diseases mortality. 13

Table S9 Sensitivity analyses of joint association of healthy behaviours and socioeconomic status on total respiratory disease mortality. 14

Table S10. Associations of socioeconomic status with total respiratory diseases mortality by sex and age. 15

Table S11. Associations of socioeconomic status with influenza and pneumonia mortality by gender and age. 16

Table S12. Associations of socioeconomic status with chronic lower respiratory diseases mortality by gender and age. 17

Table S13. The joint associations of healthy behaviours and socioeconomic status on influenza and pneumonia mortality by gender and age. 18

Table S14. The joint associations of healthy behaviours and socioeconomic status on chronic lower respiratory diseases mortality by gender and age. 19

Reference 20

Appendix method. Assessment of socioeconomic status using latent class analysis

Total household income before tax, education qualification, and employment status reflected different aspects of socioeconomic status (SES), thus we used these three parameters to generate an overall SES parameter in the UK biobank. Five groups of total household income before tax were "less than £18 000", "£18 000 to 30 999", "£31 000 to 51 999", "£52 000 to 100 000", "greater than £100 000". Seven groups of education qualification, including "College or University degree", "advanced (A) levels / advanced subsidiary (AS) levels or equivalent", "general certification of education ordinary (O) level/ the general certificate of secondary education (GCSEs) or equivalent", "the certificate of secondary education (CSEs) or equivalent", "national vocational qualification (NVQ) or higher national diploma (HND) or higher national certificate (HNC) or equivalent", "Other professional qualifications", "None of the above" (equivalent to less than high school diploma). Employment status was regrouped into two groups including employed (including those in paid employment or self-employed, retired, doing unpaid or voluntary work, or being full or part-time students), and unemployed.

Latent class analyses with different numbers of latent classes were conducted to select a reasonable model. The maximum absolute deviation between the parameter estimates in two successive iterations of the estimation procedure was set to 0.000001, which meant iteration would terminate when the difference between the parameter estimates in two successive iterations was less than 0.000001. Akaike information criterion (AIC), Bayesian information criterion (BIC), and likelihood ratio statistic G2 were used for the model selection. The mean posterior probability, which reflected the uncertainty of posterior classification, was also used for the model selection, and a higher value indicated a more acceptable uncertainty. Item-response probability was a posterior probability and was used for defining latent classes.

The G2 statistics, AIC, and BIC all continued to go down as more latent classes were added, and the decrease leveled off after the three-latent-class solution. We examined the mean posterior probabilities to facilitate the model selection. Additionally, considering we intended to compare mortality risks among individuals with different SES, sufficient sample size and events were needed among each group; however, the prevalence of "low SES" class was 7% and 4% in the four-latent-class solution and five-latent-class solution, which were relatively low. Finally, we used the three-

latent-class solution. The following table shows item-response probabilities in models with three latent classes.

Table for supplementary method. Item-response probabilities in models with three latent classes in the UK Biobank

	Latent class 1	Latent class 2	Latent class 3
Less than £18 000	0.7024	0.0007	<0.01
£18 000 to 30 999	0.2326	0.0003	0.3908
£31 000 to 51 999	0.0449	0.227	0.4285
£52 000 to 100 000	0.0095	0.5388	0.1807
Greater than £100 000	0.0106	0.2332	<0.01
College or university degree	0.1613	0.7312	0.3109
A levels/AS levels or equivalent	0.0863	0.1272	0.1374
O levels/GCSEs or equivalent	0.2257	0.0864	0.2712
CSEs or equivalent	0.0601	0.0101	0.0681
NVQ or HND or HNC or equivalent	0.0866	0.0193	0.0799
Other professional qualifications	0.0518	0.0259	0.062
None of the above	0.3282	<0.01	0.0705
Employed	0.8538	0.9564	0.9778
Unemployed	0.1462	0.0436	0.0222

As shown above, the proportion of less than £18 000 of total household income before tax, O/GCSEs level and less than high school (i.e., none of the above), and unemployment were relatively high in latent class 1, which could be defined as "low SES". The proportion of £52 000 or more of total household income before tax, college or university degree, and employment were relatively high in latent class 2, which could be defined as "high SES". £18 000-51 999 of total household income before tax, college or university degree and O/GCSEs level, and employment status were prevalent in latent class 3, which could be defined as "medium SES". The practical definitions of which were shown in the following figure.

	Employed					Unemployed				
	Less than £18 000	£18 000 to 30 999	£31 000 to 51 999	£52 000 to 100 000	Greater than £100 000	Less than £18 000	£18 000 to 30 999	£31 000 to 51 999	£52 000 to 100 000	Greater than £100 000
College or university degree	2.81	6.59	10.11	11.59	4.00	0.52	0.25	0.29	0.34	0.23
A levels/AS levels or equivalent	1.48	2.81	3.55	2.84	0.56	0.31	0.14	0.14	0.12	0.07
O levels/GCSEs or equivalent	3.94	6.17	5.98	3.42	0.46	0.68	0.31	0.24	0.15	0.06
CSEs or equivalent	0.94	1.46	1.56	0.67	0.06	0.29	0.11	0.07	0.03	0.01
NVQ or HND or HNC or equivalent	1.53	1.98	1.74	0.95	0.12	0.27	0.08	0.05	0.02	0.01
Other professional qualifications	0.96	1.52	1.34	0.85	0.16	0.10	0.04	0.04	0.02	0.01
None of the above	5.64	3.53	1.70	0.56	0.09	0.99	0.23	0.09	0.05	0.01

Figure for supplementary method. Practical definitions of high, medium, and low socioeconomic status in the three-latent-class solution (the UK Biobank).

The numbers in the cells represented the percentage of participants out of the total study population.

Red was high SES, yellow was medium SES, blue was low SES.

Table S1. The numbers (percentages) of participants with missing covariates (N=542,414)

Covariates	n	%
Race and ethnicity	2,776	0.55%
General health	3,486	0.69%
Weight loss	11,038	2.20%
Cancer	2,774	0.55%
Diabetes	2,615	0.52%
Poor psychological status	2,471	0.49%
Cardiovascular disease	2,222	0.44%
Family history	9,848	1.96%
Sleep duration	4,214	0.84%
Tea intake	2,192	0.44%
Socioeconomic status	79,393	15.80%
Healthy behaviours	33,967	6.26%

Table S2. Scoring system for healthy behaviour indices.

Variable	UK Biobank question	UK Biobank data field	Coded
Body mass index (BMI, kg/m^2) [1,2]	BMI value here is constructed from height and weight measured during the initial assessment centre visit	21001	18·5-24·9 kg/m^2=1 Others=0
Smoking [1,2]	the current/past smoking status of the participant	20116	Never=1, Others=0
Diet score [1,2]	Derived from touchscreen food frequency questionnaire	Vegetables (1289,1299); fruit (1309,1319); fish (1329,1339); type and numbers of slices/ bowls of bread (1438,1448); cereals (1458,1468); red meat intake (1369,1379, 1389); processed meat (1349)	4-7=1 0-3=0
Physical activity [1-3]	International physical activity questionnaire	864, 874, 884, 894, 904, 914	≥735 metabolic equivalent task (MET) min/week=1 <735 MET min/week=0
Alcohol intake [1,2,4]	Derived from daily and weekly alcohol questionnaire	red wine (1568, 4407); champagne/ white wine (1578, 4418); beer/ cider (1588, 4429); spirits (1598, 4440), fortified wine intake (1608, 4451); and other alcoholic drinks (5364, 4462)	Female: <5 or >15 g/day=0 Male: <5 or >30 g/day=0 Female: 5-15 g/day=1 Male: 5-30 g/day=1

Table S3. Sensitivity analyses of associations between socioeconomic status with respiratory disease mortality.

	Hazard ratio (95% CI)	
	**Unadjusted for healthy behaviours**	**Adjusted for healthy behaviours**
**After excluded participants who had an outcome event during the first 5 years of follow-up**		
High SES	1 (Reference)	1 (Reference)
Medium SES	2.23 (1.68, 2.95)	2.16 (1.63, 2.87)
Low SES	4.40 (3.31, 5.83)	4.07 (3.07, 5.40)
**Multiple imputation by chained equations to impute all missing covariates**		
High SES	1 (Reference)	1 (Reference)
Medium SES	1.06 (0.93, 1.21)	1.06 (0.93, 1.21)
Low SES	0.94 (0.81, 1.10)	0.94 (0.81, 1.10)

Notes: Models all adjusted for age, gender, race and ethnicity, general health, weight loss, diabetes, cardiovascular disease, cancer, family history, poor psychological status, sleep duration, coffee intake, and consumption of tea. 95% CI=95% confidence interval. SES= socioeconomic status.

Table S4. Associations between socioeconomic status with mortality from influenza and pneumonia and chronic lower respiratory diseases.

	Deaths/mortality (per 100 person-years)	Hazard ratio (95% CI)	
		Deaths/mortality (per 100 person-years)	**Adjusted for healthy behaviours**
**Influenza and pneumonia***			
High SES	28/0.003	64/0.01	1 (Reference)
Medium SES	128/0.005	522/0.02	1.30 (0.86, 1.96)
Low SES	196/0.018	861/0.08	2.64 (1.74, 4.00)
**Chronic lower respiratory diseases†**			
High SES	16/0.002	1 (Reference)	1 (Reference)
Medium SES	192/0.008	3.07 (1.84, 5.12)	3.05 (1.83, 5.10)
Low SES	428/0.038	7.32 (4.41, 12.16)	7.20 (4.34, 11.97)

Notes: Models all adjusted for age, gender, race and ethnicity, general health, weight loss, diabetes, cardiovascular disease, cancer, family history, poor psychological status, sleep duration, coffee intake, and consumption of tea. *Multiplicative interaction: 0.53 (95% CI: 0.12, 2.32), P=0.396; additive interaction: the synergy index=1.77 (95% CI: 0.67, 4.70). †Multiplicative interaction: 1.62 (95% CI: 0.16, 16.67), P=0.686; additive interaction: the synergy index=0.41 (95% CI: 0.17, 0.98). 95% CI=95% confidence interval. SES= socioeconomic status.

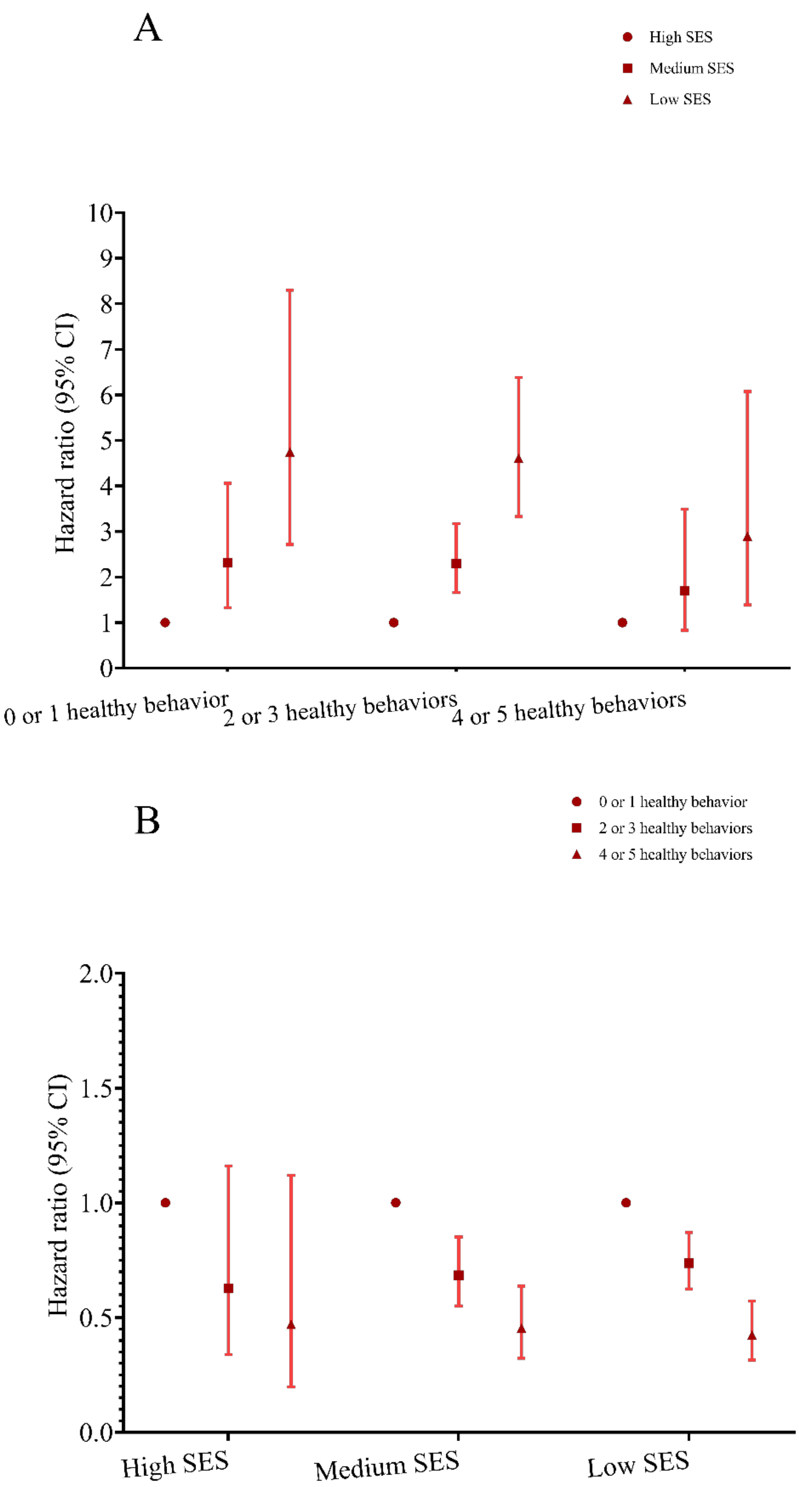

Figure S1. Associations of healthy behaviors or socioeconomic status with respiratory diseases mortality.

Models all adjusted for age, gender, race and ethnicity, general health, weight loss, diabetes, cardiovascular disease, cancer, family history, poor psychological status, sleep duration, coffee intake, and consumption of tea. Figure S1A: The reference categories for each healthy behaviours group all were high SES group. Figure S1B: The reference categories for each SES group all were no or one healthy behaviours group. 95% CI=95% confidence interval. SES= socioeconomic status.

Table S5. Associations of socioeconomic status with mortality from influenza and pneumonia and chronic lower respiratory diseases by healthy behaviors.

	0 or 1 healthy behaviour	2 or 3 healthy behaviours	4 or 5 healthy behaviours
**Influenza and pneumonia**			
High SES	1 (Reference)	1 (Reference)	1 (Reference)
Medium SES	1.40 (0.57, 3.43)	1.23 (0.74, 2.02)	1.40 (0.39, 4.97)
Low SES	3.52 (1.44, 8.58)	2.23 (1.34, 3.70)	3.50 (0.99, 12.40)
**Chronic lower respiratory diseases**			
High SES	1 (Reference)	1 (Reference)	1 (Reference)
Medium SES	3.65 (1.13, 11.80)	2.84 (1.57, 5.13)	3.39 (0.44, 26.23)
Low SES	9.57 (2.99, 30.59)	6.82 (3.79, 12.27)	4.06 (0.51, 32.52)

Models all adjusted for age, gender, race and ethnicity, general health, weight loss, diabetes, cardiovascular disease, cancer, family history, poor psychological status, sleep duration, coffee intake, and consumption of tea. The effect size was represented as hazard ratio and 95% CI. 95% CI=95% confidence interval. SES= socioeconomic status.

Table S6. Associations of healthy behaviours with respiratory disease mortality.

	Hazard ratio (95% CI)
**Total respiratory disease mortality**	
0 or 1 healthy behaviour	1 (Reference)
2 or 3 healthy behaviours	0.71 (0.63, 0.81)
4 or 5 healthy behaviours	0.44 (0.36, 0.55)
**Influenza and pneumonia**	
0 or 1 healthy behaviour	1 (Reference)
2 or 3 healthy behaviours	0.61 (0.51, 0.86)
4 or 5 healthy behaviours	0.59 (0.40, 0.88)
**Chronic lower respiratory diseases**	
0 or 1 healthy behaviour	1 (Reference)
2 or 3 healthy behaviours	0.71 (0.59, 0.86)
4 or 5 healthy behaviours	0.55 (0.17, 0.38)

Models all adjusted for socioeconomic status, age, gender, race and ethnicity, general health, weight loss, diabetes, cardiovascular disease, cancer, family history, poor psychological status, sleep duration, coffee intake, and consumption of tea. 95% CI=95% confidence interval.

Table S7. Associations of healthy behaviors with mortality from influenza and pneumonia and chronic lower respiratory diseases by socioeconomic status.

	Low SES	Medium SES	High SES
**Influenza and pneumonia**			
0 or 1 healthy behaviour	1 (Reference)	1 (Reference)	1 (Reference)
2 or 3 healthy behaviours	0.70 (0.28, 1,78)	0.74 (0.47, 1.16)	0.62 (0.43, 0.87)
4 or 5 healthy behaviours	0.44 (0.11, 1.83)	0.58 (0.29, 1.14)	0.65 (0.38, 1.09)
**Chronic lower respiratory diseases**			
0 or 1 healthy behaviour	1 (Reference)		
2 or 3 healthy behaviours	0.96 (0.26, 3.49)	0.72 (0.51, 1.03)	0.70 (0.56, 0.87)
4 or 5 healthy behaviours	0.30 (0.03, 3.06)	0.35 (0.19, 0.65)	0.20 (0.11, 0.35)

Models all adjusted for age, gender, race and ethnicity, general health, weight loss, diabetes, cardiovascular disease, cancer, family history, poor psychological status, sleep duration, coffee intake, and consumption of tea. The effect size was represented as hazard ratio and 95% CI. 95% CI=95% confidence interval. SES= socioeconomic status.

Table S8. Joint associations of healthy behaviors and socioeconomic status with respiratory diseases mortality.

	Hazard ratio (95% CI)
**Total respiratory diseases**	
Robust & 4 or 5 healthy behaviours	1 (reference)
Robust & 2 or 3 healthy behaviours	1.30 (0.63, 2.68)
Robust & 0 or 1 healthy behaviour	2.11 (0.91, 4.89)
Pre-frail & 4 or 5 healthy behaviours	1.92 (0.95, 3.90)
Pre-frail & 2 or 3 healthy behaviours	3.03 (1.56, 5.88)
Pre-frail & 0 or 1 healthy behaviour	4.55 (2.30, 9.01)
Frail & 4 or 5 healthy behaviours	3.70 (1.83, 7.47)
Frail & 2 or 3 healthy behaviours	6.22 (3.20, 12.08)
Frail1 & 0 or 1 healthy behaviour	8.32 (4.23, 16.35)
**Influenza and pneumonia**	
Robust & 4 or 5 healthy behaviours	1 (reference)
Robust & 2 or 3 healthy behaviours	1.77 (0.52, 6.00)
Robust & 0 or 1 healthy behaviour	2.67 (0.66, 10.71)
Pre-frail & 4 or 5 healthy behaviours	1.60 (0.46, 5.59)
Pre-frail & 2 or 3 healthy behaviours	2.25 (0.71, 7.16)
Pre-frail & 0 or 1 healthy behaviour	3.20 (0.96, 10.67)
Frail & 4 or 5 healthy behaviours	4.80 (1.43, 16.07)
Frail & 2 or 3 healthy behaviours	4.24 (1.33, 13.47)
Frail1 & 0 or 1 healthy behaviour	6.68 (2.04, 21.87)
**Chronic lower respiratory diseases**	
Robust & 4 or 5 healthy behaviours	1 (reference)
Robust & 2 or 3 healthy behaviours	3.07 (0.40, 23.65)
Robust & 0 or 1 healthy behaviour	3.35 (0.35, 32.26)
Pre-frail & 4 or 5 healthy behaviours	4.24 (0.56, 32.26)
Pre-frail & 2 or 3 healthy behaviours	8.70 (1.22, 62.32)
Pre-frail & 0 or 1 healthy behaviour	12.07 (1.66, 88.03)
Frail & 4 or 5 healthy behaviours	5.98 (0.78, 45.59)
Frail & 2 or 3 healthy behaviours	20.97 (2.93, 149.91)
Frail1 & 0 or 1 healthy behaviour	29.90 (4.15, 215.35)

Models all adjusted for age, gender, race and ethnicity, general health, weight loss, diabetes, cardiovascular disease, cancer, family history, poor psychological status, sleep duration, coffee intake, and consumption of tea. 95% CI=95% confidence interval. SES= socioeconomic status.

Table S9 Sensitivity analyses of joint association of healthy behaviours and socioeconomic status on total respiratory disease mortality.

	Hazard ratio (95% CI)	
	**Sensitivity analyses[1]**	**Sensitivity analyses[2]**
High SES & 4 or 5 healthy behaviours	1 (reference)	1 (reference)
High SES & 2 or 3 healthy behaviours	1.67 (0.74, 3.75)	1.69 (1.11, 2.59)
High SES & 0 or 1 healthy behaviour	2.44 (0.94, 6.30)	2.85 (1.80, 4.53)
Medium SES & 4 or 5 healthy behaviours	2.08 (0.94, 4.62)	1.07 (0.67, 1.70)
Medium SES & 2 or 3 healthy behaviours	3.65 (1.72, 7.74)	1.93 (1.28, 2.91)
Medium SES & 0 or 1 healthy behaviour	5.23 (2.41, 11.33)	2.42 (1.57, 3.73)
Low SES & 4 or 5 healthy behaviours	3.93 (1.78, 8.66)	1.16 (0.69, 1.97)
Low SES & 2 or 3 healthy behaviours	6.87 (3.24, 14.56)	1.62 (1.06, 2.47)
Low SES & 0 or 1 healthy behaviour	9.80 (4.56, 21.05)	2.36 (1.49, 3.75)

Models all adjusted for age, gender, race and ethnicity, general health, weight loss, diabetes, cardiovascular disease, cancer, family history, poor psychological status, sleep duration, coffee intake, and consumption of tea. 95% CI=95% confidence interval. SES= socioeconomic status. [1]after excluded participants who had an outcome event during the first 5 years of follow-up.

Table S10. Associations of socioeconomic status with total respiratory diseases mortality by gender and age.

	Hazard ratio (95% CI)	
	**Unadjusted for healthy behaviours**	**Adjusted for healthy behaviours**
**Male**		
High SES	1 (Reference)	1 (Reference)
Medium SES	2.84 (2.00, 4.02)	2.83 (2.00, 4.01)
Low SES	7.29 (5.14, 10.32)	7.25 (5.12, 10.26)
**Female**		
High SES	1 (Reference)	1 (Reference)
Medium SES	1.08 (0.73, 1.59)	1.08 (0.73, 1.60)
Low SES	1.64 (1.11, 2.42)	1.63 (1.11, 2.41)
**<65 years**		
High SES	1 (Reference)	1 (Reference)
Medium SES	2.07 (1.26, 3.38)	2.06 (1.26, 3.37)
Low SES	4.51 (2.77, 7.34)	4.49 (2.76, 7.31)
**≥65 years**		
High SES	1 (Reference)	1 (Reference)
Medium SES	2.32 (1.71, 3.16)	2.32 (1.70, 3.16)
Low SES	4.46 (3.26, 6.09)	4.41 (3.23, 6.03)

Models all adjusted for age, gender, race and ethnicity, general health, weight loss, diabetes, cardiovascular disease, cancer, family history, poor psychological status, sleep duration, coffee intake, and consumption of tea. 95% CI=95% confidence interval. SES= socioeconomic status.

Table S11. Associations of socioeconomic status with influenza and pneumonia mortality by gender and age.

	Hazard ratio (95% CI)	
	**Unadjusted for healthy behaviours**	**Adjusted for healthy behaviours**
**Male**		
High SES	1 (Reference)	1 (Reference)
Medium SES	1.21 (0.75, 1.94)	4.29 (2.09, 8.80)
Low SES	2.52 (1.56, 4.07)	13.86 (6.80, 28.28)
**Female**		
High SES	1 (Reference)	1 (Reference)
Medium SES	1.65 (0.70, 3.93)	1.67 (0.70, 3.97)
Low SES	3.18 (1.34, 7.55)	3.23 (1.36, 7.66)
**<65 years**		
High SES	1 (Reference)	1 (Reference)
Medium SES	1.79 (1.06, 3.02)	1.80 (1.06, 3.04)
Low SES	3.87 (2.26, 6.60)	3.89 (2.28, 6.65)
**≥65 years**		
High SES	1 (Reference)	1 (Reference)
Medium SES	0.53 (0.27, 1.02)	0.53 (0.27, 1.02)
Low SES	0.99 (0.53, 1.88)	0.99 (0.52, 1.87)

Models all adjusted for age, gender, race and ethnicity, general health, weight loss, diabetes, cardiovascular disease, cancer, family history, poor psychological status, sleep duration, coffee intake, and consumption of tea. 95% CI=95% confidence interval. SES= socioeconomic status.

Table S12. Associations of socioeconomic status with chronic lower respiratory diseases mortality by gender and age.

	Hazard ratio (95% CI)	
	**Unadjusted for healthy behaviours**	**Adjusted for healthy behaviours**
**Male**		
High SES	1 (Reference)	1 (Reference)
Medium SES	3.44 (1.80, 6.56)	3.43 (1.80, 6.53)
Low SES	7.92 (4.17, 15.07)	7.80 (4.10, 14.84)
**Female**		
High SES	1 (Reference)	1 (Reference)
Medium SES	2.49 (1.08, 5.76)	2.47 (1.07, 5.71)
Low SES	6.16 (2.69, 14.08)	6.06 (2.65, 13.85)
**<65 years**		
High SES	1 (Reference)	1 (Reference)
Medium SES	4.31 (2.10, 8.85)	4.29 (2.09, 8.80)
Low SES	14.05 (6.89, 28.67)	13.86 (6.80, 28.28)
**≥65 years**		
High SES	1 (Reference)	1 (Reference)
Medium SES	1.27 (0.62, 2.63)	1.28 (0.62, 2.64)
Low SES	2.06 (1.01, 4.21)	2.04 (1.00, 4.17)

Models all adjusted for age, gender, race and ethnicity, general health, weight loss, diabetes, cardiovascular disease, cancer, family history, poor psychological status, sleep duration, coffee intake, and consumption of tea. 95% CI=95% confidence interval. SES= socioeconomic status.

Table S13. The joint associations of healthy behaviours and socioeconomic status on influenza and pneumonia mortality by gender and age.

	Hazard ratio (95% CI)
**Male**	
High SES & 4 or 5 healthy behaviours	1 (reference)
High SES & 2 or 3 healthy behaviours	1.52 (0.34, 6.70)
High SES & 0 or 1 healthy behaviour	3.00 (0.60, 14.95)
Medium SES & 4 or 5 healthy behaviours	0.75 (0.14, 4.09)
Medium SES & 2 or 3 healthy behaviours	2.20 (0.54, 9.03)
Medium SES & 0 or 1 healthy behaviour	2.31 (0.52, 10.18)
Low SES & 4 or 5 healthy behaviours	4.53 (1.00, 20.62)
Low SES & 2 or 3 healthy behaviours	3.83 (0.93, 15.81)
Low SES & 0 or 1 healthy behaviour	6.22 (1.46, 26.48)
**Female**	
High SES & 4 or 5 healthy behaviours	1 (reference)
High SES & 2 or 3 healthy behaviours	2.20 (0.26, 18.84)
High SES & 0 or 1 healthy behaviour	NA
Medium SES & 4 or 5 healthy behaviours	3.06 (0.39, 24.02)
Medium SES & 2 or 3 healthy behaviours	2.01 (0.27, 15.12)
Medium SES & 0 or 1 healthy behaviour	6.06 (0.77, 47.85)
Low SES & 4 or 5 healthy behaviours	5.30 (0.68, 41.14)
Low SES & 2 or 3 healthy behaviours	4.79 (0.65, 35.45)
Low SES & 0 or 1 healthy behaviour	7.47 (0.95, 58.63)
**<65 years**	
High SES & 4 or 5 healthy behaviours	1 (reference)
High SES & 2 or 3 healthy behaviours	1.51 (0.33, 6.85)
High SES & 0 or 1 healthy behaviour	2.71 (0.49, 14.90)
Medium SES & 4 or 5 healthy behaviours	1.87 (0.40, 8.82)
Medium SES & 2 or 3 healthy behaviours	2.75 (0.67, 11.34)
Medium SES & 0 or 1 healthy behaviour	4.56 (1.05, 19.88)
Low SES & 4 or 5 healthy behaviours	4.26 (0.88, 20.64)
Low SES & 2 or 3 healthy behaviours	5.25 (1.26, 21.83)
Low SES & 0 or 1 healthy behaviour	13.06 (3.06, 55.73)
**≥65 years**	
High SES & 4 or 5 healthy behaviours	1 (reference)
High SES & 2 or 3 healthy behaviours	2.04 (0.25, 16.35)
High SES & 0 or 1 healthy behaviour	2.13 (0.19, 23.52)
Medium SES & 4 or 5 healthy behaviours	0.77 (0.09, 6.44)
Medium SES & 2 or 3 healthy behaviours	1.03 (0.14, 7.53)
Medium SES & 0 or 1 healthy behaviour	1.07 (0.13, 8.75)
Low SES & 4 or 5 healthy behaviours	2.38 (0.32, 17.97)
Low SES & 2 or 3 healthy behaviours	1.84 (0.25, 13.34)
Low SES & 0 or 1 healthy behaviour	1.50 (0.19, 11.66)

Models all adjusted for age, gender, race and ethnicity, general health, weight loss, diabetes, cardiovascular disease, cancer, family history, poor psychological status, sleep duration, coffee intake, and consumption of tea. Nine joint groups were presented in the following order: High SES & four or five healthy behaviours, High SES & two or three healthy behaviours, High SES & no or one healthy behaviour, Medium SES & four or five healthy behaviours, Medium SES & two or three healthy behaviours, Medium SES & no or one healthy behaviour, Low SES & four or five healthy behaviours, Low SES & two or three healthy behaviours, Low SES & no or one healthy behaviour. 95% CI=95% confidence interval. SES = socioeconomic status.

Table S14. The joint associations of healthy behaviours and socioeconomic status on chronic lower respiratory diseases mortality by gender and age.

	Hazard ratio (95% CI)
**Male**	
High SES & 4 or 5 healthy behaviours	1 (reference)
High SES & 2 or 3 healthy behaviours	1.35 (0.17, 11.01)
High SES & 0 or 1 healthy behaviour	1.55 (0.14, 17.17)
Medium SES & 4 or 5 healthy behaviours	2.01 (0.24, 16.73)
Medium SES & 2 or 3 healthy behaviours	4.97 (0.69, 35.76)
Medium SES & 0 or 1 healthy behaviour	5.07 (0.68, 37.66)
Low SES & 4 or 5 healthy behaviours	2.98 (0.35, 25.60)
Low SES & 2 or 3 healthy behaviours	10.97 (1.53, 78.87)
Low SES & 0 or 1 healthy behaviour	13.67 (1.88, 99.40)
**Female**	
High SES & 4 or 5 healthy behaviours	1 (reference)
High SES & 2 or 3 healthy behaviours	NA
High SES & 0 or 1 healthy behaviour	NA
Medium SES & 4 or 5 healthy behaviours	NA
Medium SES & 2 or 3 healthy behaviours	NA
Medium SES & 0 or 1 healthy behaviour	NA
Low SES & 4 or 5 healthy behaviours	NA
Low SES & 2 or 3 healthy behaviours	NA
Low SES & 0 or 1 healthy behaviour	NA
**<65 years**	
High SES & 4 or 5 healthy behaviours	1 (reference)
High SES & 2 or 3 healthy behaviours	NA
High SES & 0 or 1 healthy behaviour	NA
Medium SES & 4 or 5 healthy behaviours	NA
Medium SES & 2 or 3 healthy behaviours	NA
Medium SES & 0 or 1 healthy behaviour	NA
Low SES & 4 or 5 healthy behaviours	NA
Low SES & 2 or 3 healthy behaviours	NA
Low SES & 0 or 1 healthy behaviour	NA
**≥65 years**	
High SES & 4 or 5 healthy behaviours	1 (reference)
High SES & 2 or 3 healthy behaviours	1.48 (0.18, 12.30)
High SES & 0 or 1 healthy behaviour	0.97 (0.06, 15.58)
Medium SES & 4 or 5 healthy behaviours	1.29 (0.17, 10.01)
Medium SES & 2 or 3 healthy behaviours	1.63 (0.23, 11.81)
Medium SES & 0 or 1 healthy behaviour	2.25 (0.30, 16.92)
Low SES & 4 or 5 healthy behaviours	0.76 (0.09, 6.23)
Low SES & 2 or 3 healthy behaviours	2.85 (0.40, 20.44)
Low SES & 0 or 1 healthy behaviour	3.79 (0.52, 27.66)

Models all adjusted for age, gender, race and ethnicity, general health, weight loss, diabetes, cardiovascular disease, cancer, family history, poor psychological status, sleep duration, coffee intake, and consumption of tea. Nine joint groups were presented in the following order: High SES & four or five healthy behaviours, High SES & two or three healthy behaviours, High SES & no or one healthy behaviour, Medium SES & four or five healthy behaviours, Medium SES & two or three healthy behaviours, Medium SES & no or one healthy behaviour, Low SES & four or five healthy behaviours, Low SES & two or three healthy behaviours, Low SES & no or one healthy behaviour. SES = socioeconomic status. 95% CI=95% confidence interval.

Reference

1. Bountziouka, V.; Musicha, C.; Allara, E.; Kaptoge, S.; Wang, Q.; Angelantonio, E.D.; Butterworth, A.S.; Thompson, J.R.; Danesh, J.N.; Wood, A.M., et al. Modifiable traits, healthy behaviours, and leukocyte telomere length: a population-based study in UK Biobank. *The Lancet. Healthy longevity* **2022**, *3*, e321-e331, doi:10.1016/s2666-7568(22)00072-1.
2. Yang, G.; Cao, X.; Li, X.; Zhang, J.; Ma, C.; Zhang, N.; Lu, Q.; Crimmins, E.M.; Gill, T.M.; Chen, X., et al. Association of Unhealthy Lifestyle and Childhood Adversity With Acceleration of Aging Among UK Biobank Participants. *JAMA network open* **2022**, *5*, e2230690, doi:10.1001/jamanetworkopen.2022.30690.
3. Fan Mengyu, Lyu Jun, He Pingping. Chinese guidelines for data processing and analysis concerning the International Physical Activity Questionnaire. Chinese Journal of Epidemiology. 2014;35(08):961-964.
4. Zhang, Y.B.; Chen, C.; Pan, X.F.; Guo, J.; Li, Y.; Franco, O.H.; Liu, G.; Pan, A. Associations of healthy lifestyle and socioeconomic status with mortality and incident cardiovascular disease: two prospective cohort studies. *BMJ (Clinical research ed.)* **2021**, *373*, n604, doi:10.1136/bmj.n604.
